# Postpartum Psychosis and Maternal Filicide- Case Report and Literature Review

**DOI:** 10.1192/j.eurpsy.2024.1688

**Published:** 2024-08-27

**Authors:** O. Ali, S. Islam, H. Raai, L. Troneci

**Affiliations:** ^1^Psychiatry, SBH Health System; ^2^CUNY School of Medicine, New York, United States

## Abstract

**Introduction:**

Postpartum period is defined as the 12 weeks following the birth of a child as per ACOG guidelines. This period is crucial for women to physically and emotionally adapt to major changes in their lives. If concerns are not addressed properly it can result in fatal outcomes such as filicide and suicide in context of untreated mental illness with postpartum onset. Postpartum psychosis is considered a psychiatric emergency and literature shows that up to 4.5% of patients with depressive symptoms with psychosis commit filicide. However, postpartum psychosis is not recognized as a formal psychiatric disorder in DSM-5, leading to a delay in identification and treatment of the condition in a timely fashion.

**Objectives:**

The primary purpose of the case report is to inform the clinical picture and the legal implications associated with postpartum psychosis, a poorly understood and underdiagnosed psychiatric illness and to emphasize the importance of considering other psychiatric illnesses with peripartum onset that affect maternal and pediatric population wellbeing.

**Methods:**

A comprehensive review of literature using databases, such as PubMed and Google Scholar as well as observation of the patient in the Emergency Department by the psychiatry team.

**Results:**

We present the case of a female in her 20s, mother of two toddlers, with a history of PTSD and postpartum depression, who was brought to our Emergency Department for stabbing her children in the context of a psychotic episode. The patient endorsed persecutory delusions and religious preoccupation, stating that she was experiencing “demonic energy inside” and that demons were speaking through her sons. Upon further assessment, it was noted that symptom onset was during the peripartum period, initially with depressed mood, and later with psychotic features. Organic causes of psychosis were ruled out with an extensive workup. Patient was transferred to an inpatient forensic unit for further stabilization. From a legal perspective, literature review shows that mothers may face the death penalty in the US in contrast with other countries such as England for instance. In the context of the current case, the plausible diagnoses are MDD with psychotic features or the first psychotic episode with peripartum onset that was left untreated resulting in a fatal health and legal outcome.

**Conclusions:**

As postpartum psychosis is not currently recognized as an independent diagnosis under the DSM-5, further attention is warranted for such critical psychiatric condition that afflicts the lives and well-being of the maternal and pediatric populations globally. Postpartum psychosis affects mothers despite their past psychiatric history, socioeconomic status, educational level, and supportive network. Thus, it is essential to target proper and timely identification of symptoms and address those to prevent filicide and maternal suicide.

**Disclosure of Interest:**

None Declared

